# Posterior lumbar interbody fusion graft penetrated the lumbar thecal sac in a patient with rheumatoid arthritis: A case report

**DOI:** 10.1016/j.ijscr.2020.01.017

**Published:** 2020-01-23

**Authors:** Dong-Ju Lim, Joon-Ho Lee

**Affiliations:** Department of Orthopaedic Surgery, Seoul Spine Institute, Sanggyepaik Hospital, College of Medicine, Inje University, Republic of Korea

**Keywords:** RA, rheumatoid arthritis, HIVD, herniated intervertebral disc, MTX, methotrexate, MRI, magnetic resonance imaging, CT, computed tomography, PLIF, posterior lumbar interbody fusion, Rheumatoid arthritis, Pseudoarthrosis, Graft material, Cauda equina syndrome, Revision spine surgery

## Abstract

**Introduction:**

Intradural foreign bodies have been reported to be associated with disc material, tumors, and bullets following spinal gunshot injuries. In this report, we describe a case of non-union with minor trauma that caused interbody bone graft material to migrate into the intrathecal area in a patient with RA.

**Presentation of case:**

We present the case of a 65-year-old woman visited an outpatient clinic of our hospital after experiencing progressive lower extremity weakness, and voiding and defecation difficulty after fell down several times in the past. She had a history of two spinal decompression with fixation surgeries due to spinal stenosis with a herniated intervertebral disc. She was prescribed steroids and methotrexate for the RA. The results of MRI and CT demonstrated an intradural bone graft material migration with cauda equina syndrome after revision lumbar stenosis surgery. Calcified material protruded to the intracanal area and compressed the cauda equina fiber. After the removal of fragments operation, she recovered from cauda equina symptoms. A follow-up examination two years postoperatively revealed clinical resolution of cauda equina symptoms and a return to partial walking with a cane.

**Discussion:**

The patient had a minor or major trauma, such as a fall, after the revision surgery. After that trauma, the patient presented with some dural injury, kyphotic position, or non-union state causing the dural penetration of the interbody fusion material.

**Conclusion:**

The first report describing displaced PLIF graft material that penetrated the dural sac and caused cauda equina symptoms in a patient with RA. Establishing strategies to minimize these complications is indicated when treating degenerative lumbar spine conditions in patients with RA.

## Highlights

• Case of non-union with minor trauma that caused interbody bone graft material to migrate into the intrathecal area in a patient with RA.
• Additional L5 laminectomy and S1 pedicle screw insertion with bone graft material removal was applied. And neurologic deficit was recovered.
• Our hypothesis was dural injury occurred after trauma, kyphotic position or non-union state causing the dural penetration of the fusion material.

## 1 Introduction

Intradural foreign bodies have been reported to be associated with disc material, tumors, and bullets following spinal gunshot injuries. Several studies have noted a higher rate of complications when implants are used in patients with rheumatoid arthritis (RA) owing to the poor quality of the bone and formation process caused by various factors, including anti-RA medication, circulating cytokines, disease activity, and reduced physical activity. In this report, we describe a case of non-union with minor trauma that caused interbody bone graft material to migrate into the intrathecal area with cauda equine syndrome in a patient with RA.

This article has been written according to SCARE criteria as described by Agha et al. for the SCARE group. ‘The SCARE 2018 Statement: Updating consensus Surgical CAse REeport (SCARE) guidelines. International Journal of Surgery 2018’ [1].

## 2 Presentation of case

### 2.1 Patient history and presenting features

A 65-year-old woman visited an outpatient clinic of our hospital after experiencing progressive illness, lower extremity weakness, and voiding and defecation difficulty for two weeks. She had fallen several times in the past. She had a history of two spinal decompression surgeries and posterolateral fusion with pedicle screw fixation surgery due to spinal stenosis with a herniated intervertebral disc (HIVD) at the L1-L5 level, which was performed in our hospital. She had undergone thyroidectomy due to thyroid cancer and had a medical history of RA. She was prescribed steroids and methotrexate (MTX) for the RA.

### 2.2 Radiologic investigation and physical examination

**Fig. 1:**
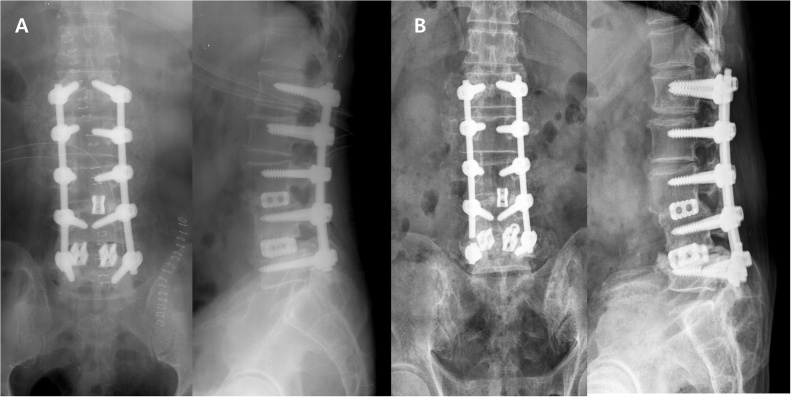
**A**. Decompression and fixation with pedicle screws were performed due to spinal stenosis in a patient with a herniated intervertebral disc. **B**. Both L5 pedicle screws and rods are broken and local kyphosis occurred 5 years after the surgery.

Plain radiography revealed that both L5 pedicle screws and rods were broken and that local kyphosis had occurred below the lower-instrumented vertebrae level. In the previous operation, interbody fusion had been performed with a local autograft, allograft, and metal cage (Fig. 1).

**Fig. 2:**
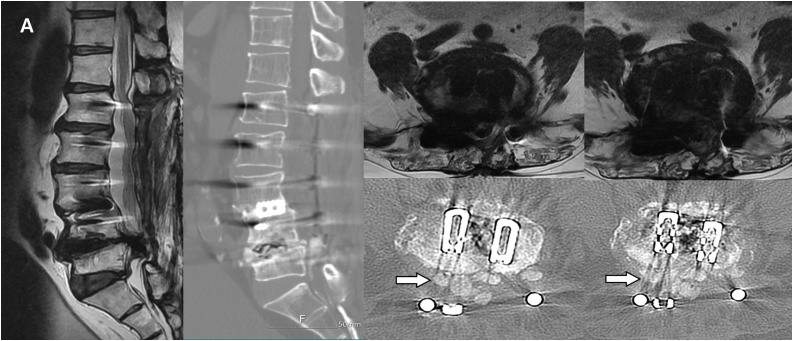
**A**. T2-weighted magnetic resonance imaging shows low-signal material at the L4-5 level. **B**. Three-dimensional computed tomography in the sagittal view shows local kyphosis and L5 body collapse, with a bone-like lesion at the L4-5 level. **C, D**. Calcified material protruded to the intracanal area and compressed the cauda equina fiber.

The results of magnetic resonance imaging (MRI) and computed tomography (CT) demonstrated an intradural foreign body in the spinal canal at the L4-5 level (Fig. 2A, B). There was an obscure margin between the ventral dura and an abnormal foreign body around the L4-L5 disc area. A pathological foreign body appeared as calcification in the canal with a marked collapse of the dural sac, which showed severe canal stenosis with space-occupying material at the L4-L5 level (Fig. 2C). There was a weakness in both the legs and ankles (power 0-2/5) and decreased sensation below the L4 dermatome level. Sphincter tone decreased, and saddle anesthesia observed.

### 2.3 Treatment and outcome

**Fig. 3:**
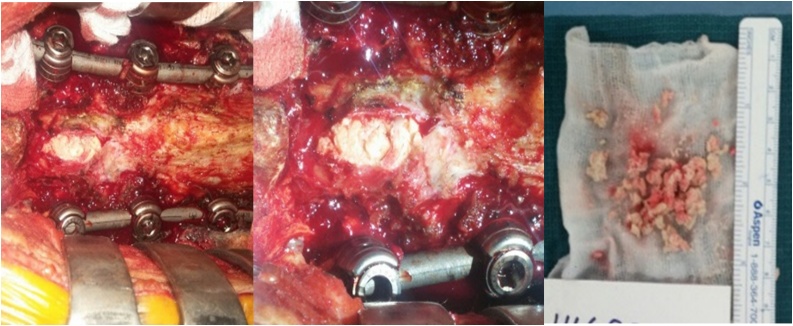
**A**. An intraoperative finding of a foreign body in the incised dural sac. **B**. The foreign body is a homogenous material, like powdered calcium.

The authors performed standard procedures, including removal of foreign body, extensive decompression, and fusion for spinal stenosis accompanied by cauda equina syndrome. The screws and rods were the same products from the initial operation and extended fixation was done. The patient was treated with an additional L5 laminectomy and S1 pedicle screw insertion. Adhesions between the dura and the ligament caused fixation of the dural sac and a hard intradural mass was palpable, so the decision was made to open the dura and explore the cauda equina fiber. Upon opening the dura, multiple fragments of bone graft material were seen in the spinal canal, displacing the roots centrally and peripherally (Fig. 3A). The fragments were removed, and an orifice was not detected on the anterior dura. The incised posterior dura was sutured. The dura was closed primarily, and nerve root decompression was ensured. Postoperatively, the patient recovered smoothly, and bladder sensations started recovering immediately on the day of the operation. A histopathologist diagnosed the foreign material as an amorphous homogenous material, like calcium powder. A follow-up examination 2 years postoperatively revealed clinical resolution of cauda equina symptoms and a return to partial walking with a cane.

## 3 Discussion

In the spine, RA more frequently involves the cervical vertebrae than the lumbar vertebrae [2,3], but there have been several reports in which RA involved the lumbar spine [[4], [5], [6]]. Crawford et al. [7] reported a high rate of complications in patients who underwent lumbar fusion; the rate of non-union was 10.5% in patients with RA. Moreover, there is also a high incidence of infection after spine surgery in patients with RA.

The most typical intradural foreign bodies described in the literature are shotgun bullets, and cases of glass or metal debridement have been presented [[8], [9], [10], [11]]. Intraradicular disc herniation, as a special type of intradural disc herniation, is an extremely rare neurosurgical entity first described by Barberá et al. in 1984 [12].

We consider the migration of the bone graft material, implanted in the previous surgery, into the intrathecal area by minor traumatic event is the origin of the calcified materials found within the spinal canal. An explanation for dural perforation from disc herniation is not clear, although there are several known factors that could contribute to it, such as a congenital narrowing of the spinal canal with less epidural space; adhesions between the annulus fibrosus, posterior longitudinal ligament, and/or dura mater; and congenital and iatrogenic tears of the dura mater [[12], [13], [14]].

The clinical outcomes for patients with RA who undergo lumbar spine surgery are relatively unknown. However, there is a high rate of reported complications following posterior lumbar interbody fusion (PLIF) for patients with RA, including collapse of adjacent vertebra (57%), instability at the adjacent level (43%), migration of pedicle screws (29%), collapse of grafted bone (14%), decubitus, and infection (14%) [6]. In this case, the L4-5 interbody graft material was displaced posteriorly, and the collapse of the upper-end plate of the L5 body and L5 screw loosening was observed. In another study, complications were reported as symptomatic adjacent segmental disease (70%), pseudarthrosis (19%), adjacent vertebral fracture (11%), screw loosening (26%), infection (7%) [15].

In the mutilating type of RA with severe osteoporosis, PLIF in combination with a long fixation system and augmentation of the vertebral bodies might be needed. Inaoka et al. [16] performed PLIF for seven patients with RA and reported that the collapse of the graft occurred in one patient, migration of the pedicle screw in two, instability of the adjacent level in three, and collapse of adjacent vertebrae in four. In cases of instrumentation surgery for lumbar canal stenosis in RA, postoperative orthosis treatment is stricter and longer than usual due to osteoporosis, which may cause instrumentation failure or pathological fracture after surgery [17].

Immunosuppressive drugs, disease-modifying anti-rheumatic drugs, and steroids used to treat patients with RA adversely affect new bone formation. MTX causes osteoporosis, stress fractures, and bone pain when used in high doses to treat cancer. MTX has been shown to cause decreased osteoblast proliferation in human osteoblasts. Its effects on people with rheumatic diseases are more controversial because most patients using MTX have other risk factors for bone fractures. In addition, these patients may be taking other medications that affect bone metabolism. In addition, low doses of MTX are generally used for patients with RA rather than in patients with cancer. A recent study [18] suggests no adverse effects of low-dose MTX on markers of bone formation, turnover, and mass, although a study reported that corticosteroids and cancer chemotherapeutic agents generally affect bone adversely and increase fracture risk and bone metabolism [19].

Clinically, glucocorticoid-induced bone loss develops rapidly. It has generally been thought that glucocorticoid-induced osteoporosis results from the suppressed bone formation [20]. Furthermore, a study on the relationship between steroid usage and clinical outcomes following craniovertebral junctional spine surgery reported significantly smaller improvements in outcomes compared to patients not taking steroids and those taking prednisone dosages less than 7.5 mg [18].

Our hypothesis was that the patient had a minor or major trauma, such as a fall, after the revision surgery. After that trauma, the patient presented with some dural injury, kyphotic position, or non-union state causing the dural penetration of the interbody fusion material.

## 4 Conclusion

This case is the first report describing displaced PLIF graft material that penetrated the dural sac and caused cauda equina symptoms in a patient with RA. Establishing strategies to minimize these complications is indicated when treating degenerative lumbar spine conditions in patients with RA.

## Sources of funding

This work was supported by a grant from Research of Inje University.

## Ethical approval

This case report has been approved by institutional review board (IRB) of Sanggyepaik Hospital, Inje University with waived informed consent to the patient. (SGPAIK 2019-03-012).

## Consent

The head of our medical team has taken responsibility that exhaustive attempts have been made to contact the family of all of the patients and that the paper has been sufficiently anonymised not to cause harm to the patients or their families. A signed document to this effect is available if required by the Editor in Chief.

## Author contribution

Dong-Ju, Lim - study concept, design.

Joon-Ho, Lee - data collection, data analysis or interpretation, writing the paper.

## Registration of research studies

Institutional review board of Sanggyepaik Hospital, Inje University with waived informed consent. (SGPAIK 2019-03-012).

I will send the documents if you want.

## Guarantor

Dong-Ju, Lim.

## Provenance and peer review

Not commissioned, externally peer-reviewed.

## Declaration of Competing Interest

The authors have no conflict of interests to declare.
